# Obesity associates with vasomotor symptoms in postmenopause but with physical symptoms in perimenopause: a cross-sectional study

**DOI:** 10.1186/s12905-017-0487-7

**Published:** 2017-12-08

**Authors:** Seul Koo, Younjhin Ahn, Joong-Yeon Lim, Juhee Cho, Hyun-Young Park

**Affiliations:** 1 Division of Cardiovascular Diseases, Center for Biomedical Sciences, Korea National Institute of Health, 187 Osongsaengmyeng 2-Ro, Osong-eup, Heungdeok-gu, Cheongju-si, Chungcheongbuk-Do 28160 Republic of Korea; 2 Division of Rare Diseases, Center for Biomedical Sciences, Korea National Institute of Health, 187 Osongsaengmyeng 2-Ro, Osong-eup, Heungdeok-gu, Cheongju-si, Chungcheongbuk-Do 28160 Republic of Korea; 3 Department of Clinical Research Design and Evaluation, SAIHST, Sungkyunkwan University, Seoul, Republic of Korea; 4 Departments of Epidemiology and Health, Behavior and Society, Johns Hopkins Bloomberg School of Public Health, Baltimore, USA

**Keywords:** Obesity, Body mass index, Menopause, Menopausal symptom

## Abstract

**Background:**

Middle-aged women experience various menopausal symptoms during the menopause. These symptoms can affect their quality of life and health. Several epidemiological studies reported that obesity associates with menopausal symptoms. The aim of this cross-sectional study was to examine the associations between obesity and multiple menopausal symptoms at different stages of menopause in middle-aged Korean women.

**Methods:**

The study population included women aged 44–56 years who visited a tertiary referral hospital for medical check-ups between November 2012 and March 2013 and were free from serious illness, could comprehend a questionnaire. The Menopause-Specific Quality of Life (MENQOL) questionnaire was used to assess the prevalence of menopausal symptoms. Overweight and obesity were defined as body mass index (BMI) of 23–24.9 and ≥25 kg/m^2^, respectively.

**Results:**

Of the 2204 middle-aged women, 929 met the eligibility criteria. Of these, 533 (57.4%) and 396 (42.6%) were in perimenopause and postmenopause, respectively. In perimenopause, obese women were significantly more likely to have moderate/severe physical symptoms (MENQOL domain score ≥ 5) than normal or overweight women. In postmenopause, obese women were significantly more likely to have moderate/severe vasomotor symptoms. Multiple linear regression with adjustment for confounders showed that relative to normal weight, obesity in perimenopause and postmenopause associated independently with physical symptoms (beta coefficient = 0.35; *P* = 0.023) and vasomotor symptoms (beta coefficient = 0.68; *P* = 0.003), respectively. Overweight did not associate with menopausal symptoms. BMI did not associate significantly with psychosocial or sexual symptoms at either stage of menopause.

**Conclusions:**

Obese women had more frequent menopausal symptoms than normal or overweight women but the associated menopausal symptom differed depending on the menopausal stage. Further studies are required to confirm this result and identify the underlying mechanisms.

**Electronic supplementary material:**

The online version of this article (doi:10.1186/s12905-017-0487-7) contains supplementary material, which is available to authorized users.

## Background

Menopause is a period in a woman’s life that associates with marked hormonal and metabolic changes that can cause a wide range of sometimes bothersome menopausal symptoms [1]. The typical symptoms of menopause include vasomotor, psychological, physical, and sexual symptoms. These symptoms can differ in severity, duration, and nature depending on stage of menopause, lifestyle factors, and race/ethnicity [2, 3]. The period from peri- to postmenopause not only involves hormonal and metabolic changes, but also associates with changes in body composition, including an increase in body fat and weight gain [4]. This suggests that menopause may partly contribute to the rising prevalence of obesity among middle-aged women. However, the inter-relationships between weight, menopausal process, aging, and hormone levels remain poorly understood [4, 5]. Several studies assessing the relationship between body mass index (BMI) or body fat and menopausal symptoms yielded inconsistent results [6–8]. Moreover, earlier cross-sectional studies conducted in Australia and Turkey failed to detect significant associations between BMI and menopausal symptoms [9, 10], whereas longitudinal studies suggest that women with higher BMI or body fat have a higher risk of vasomotor symptoms and other menopausal symptoms [8, 11–13]. Recent intervention trials suggest that weight loss in overweight or obese women may improve the hot flashes that are the most common symptom of menopause [14, 15]. However, the relationship between obesity and menopausal symptoms at various stages of menopausal process remains poorly understood. Moreover, most of studies on the associations between obesity and menopausal symptoms only focused on the vasomotor symptoms such as hot flashes, despite the fact that women undergo multiple diverse symptoms during transition [6, 16–19].

The objectives of this study were to investigate the associations between BMI and a large range of menopausal symptoms at different stages of menopause in Korean women.

## Methods

This cross-sectional study was approved by the Internal Review Board of Kangbuk Samsung Hospital (IRB no. KBC12156). All subjects provided written informed consent to participate in the study.

### Study population

The study population included eligible women who underwent a medical health check-up between November 2012 and March 2013 at the two healthcare centers of Kangbuk Samsung Hospital located in Seoul and Suwon, South Korea [20]. These centers has approximately 63,000 visitors each year, most of the visitors are employee from various companies for either annual or biennial health checkup. The total number of visitors during the study period was 42,965 people, among them 4209 were eligible women (44–56 years old). Participants who did not consent to be included in the study and who were unable to comprehend a questionnaire were excluded in screening stage. In total, 2204 women were recruited and consent in this study. Of these, 1275 were excluded because they were premenopausal (*n* = 809), had serious illnesses (e.g., breast cancer, *n* = 43) and/or who had used hormone replace therapy (*n* = 132), and who had missing data on menopausal symptoms or BMI or covariate variables (*n* = 291). Finally, 929 subjects were included in present analysis (See Additional file 1).

### Data collection and definitions

The following socio-demographic and lifestyle data were obtained by providing each subject with a self-administered questionnaire: age (years), education (“high school graduate or less” or “some college or more”), monthly household income (“<4,000,000 KRW (Korean won) won” or “≥4,000,000 KRW”), employment status (“employed” or “unemployed”), marital status (“yes” or “no” to the question, “are you living with a spouse?”) and parity (“1–2” or “3 or more”, depending on the number of deliveries), current smoking status (“yes” or “no”), alcohol intake (“never” or “ever”), and physical activity (“low”, “moderate”, or “high”, as determined using the short format of the International Physical Activity Questionnaire [21]).

During the same visit, blood was taken and serum levels of total cholesterol, triglycerides, high density lipoprotein (HDL)-cholesterol, and fasting blood glucose were analyzed. Height, weight, and waist circumference were measured.

Menopausal symptoms were assessed by using the Korean translation version (See Additional file 2) of the original Menopause-Specific Quality of Life (MENQOL) questionnaire [22]. MENQOL is a widely used tool for evaluating menopausal symptoms and consist of 29-item validated instrument that assesses four domains [23, 24]: vasomotor (“hot flushes or flashes”, “night sweats”, and “sweating”), psychosocial (e.g., “feeling anxious or nervous”, “experiencing poor memory”, “feeling depressed, down or blue”, and “feeling of wanting to be alone”), physical (e.g., “flatulence (wind) or gas pains”, “aching in muscles and joints”, “difficulty sleeping”, and “weight gain”), and sexual (“change in sexual desire”, “vaginal dryness during intercourse”, and “avoiding intimacy”). All items are scored as follows: participant is asked whether she currently experiences the symptoms and, if she answers “yes”, then she is asked how bothered she is by the symptoms using a 7-point Likert scale where 0 indicates not at all bothered and 6 indicates extremely bothered by symptom.

The menopausal status of subjects was defined on basis of the STRAW criteria [25]. Perimenopause encompasses both early transition (a gap in normal menstrual period of longer than 7 days) and late transition (≥2 skipped menstrual cycles or no menses for ≥60 days). Postmenopause is defined as no menses for at least 12 months.

BMI was calculated by dividing the weight (in kilograms) by the height (in meters) squared. The study population was classified into three BMI groups on the basis of World Health Organization (WHO) Asian BMI criteria [26]: normal (BMI = 18.5–22.9 kg/m^2^), overweight (BMI = 23–24.9 kg/m^2^), and obese (BMI ≥ 25 kg/m^2^).

### Data analysis

Continuous variables are expressed as mean ± standard deviation while categorical variables are expressed as n(%). The subjects in perimenopause and postmenopause were divided into three subgroups on the basis of their BMI. The BMI subgroups in perimenopause or postmenopause group were compared in terms of continuous variables using analysis of variance with Scheffe’s multiple comparison test and in terms of categorical variables using Chi-squared test.

For each of 29 MENQOL items, 7-point Likert scale is converted the score ranging from 1 (no symptom) to 8 (experiencing symptom and extremely bothersome).

The prevalence of each of 29 individual component symptoms in MENQOL was determined by considering the scores of 1 and 2–8 to mean “no symptom” and “experienced symptom”, respectively. MENQOL domain scores that were <5 were considered to indicate “not bothersome symptoms” while scores of ≥5 were regarded to indicate ‘moderate/severe symptoms’. Multiple linear regression analysis was performed to estimate the association between overweight and obesity and menopausal symptoms in perimenopause and postmenopause relative to normal weight. The multiple linear regression model was also used to adjust for age, education level, parity, marital status, and physical activity. All statistical analyses were conducted using SAS software, version 9.2 (SAS Institute, Inc., Cary, NC). *P*-values of <0.05 were considered to indicate statistical significance. All statistical tests were two-tailed.

## Results

Of the 929 eligible women, 533 (57.4%) were perimenopausal and 396 (42.6%) were postmenopausal. With regard to the 533 perimenopausal women, 293 (55.0%), 127 (23.8%), and 113 (21.2%) were normal, overweight, and obesity, respectively. With regard to postmenopausal women, 184 (46.5%), 94 (23.7%), and 118 (29.8%) were normal, overweight, and obesity, respectively. The characteristics of normal, overweight, and obesity subgroups in perimenopausal and postmenopausal groups are shown in Table 1. The mean age of women in perimenopause and postmenopause was 48.6 ± 2.8 and 52.0 ± 3.1 years old, respectively. In both perimenopausal and postmenopausal groups, the obese women had significantly higher levels of triglycerides and fasting blood glucose and lower HDL-cholesterol levels than normal women. In perimenopausal group, obese women also had significantly higher total cholesterol levels than normal women.

**Table 1:** Demographic and clinical characteristics of the normal, overweight, and obese perimenopausal and postmenopausal subjects

Characteristic	Perimenopause				Postmenopause			
	Normal	Overweight	Obesity	*p*-value	Normal	Overweight	Obesity	*p*-value
	(*n* = 293)	(*n* = 127)	(*n* = 113)		(*n* = 184)	(*n* = 94)	(*n* = 118)	
Age, years	48.4 ± 2.7	48.6 ± 2.8	48.8 ± 2.9	0.438	52.0 ± 3.0	51.8 ± 3.0	52.2 ± 3.4	0.573
Education								
High school graduate or less	91 (31.1)	57 (44.9)	56 (49.6)	0.001	91 (49.5)	51 (54.3)	70 (59.3)	0.242
Some college or more	202 (68.9)	70 (55.1)	7 (50.4)		93 (50.5)	43 (45.7)	48 (40.7)	
Household income/monthly								
<4,000,000 KRW	34 (13.9)	17 (15.9)	16 (16.5)	0.797	24 (16.1)	21 (28.8)	29 (30.2)	0.017
≥4,000,000 KRW	210 (86.1)	90 (84.1)	81 (83.5)		125 (83.9)	52 (71.2)	67 (69.8)	
Employment status, employed	122 (48.2)	56 (52.3)	49 (49.5)	0.775	66 (41.8)	40 (54.8)	52 (54.7)	0.064
Living with a spouse, yes	282 (96.3)	118 (92.9)	103 (91.2)	0.098	174 (94.6)	83 (88.3)	103 (87.3)	0.060
Parity								
1–2	244 (83.3)	107 (84.3)	84 (74.3)	0.077	144 (78.3)	58 (61.7)	89 (75.4)	0.011
3 or more	49 (16.7)	20 (15.8)	29 (25.7)		40 (21.7)	36 (38.3)	29 (24.6)	
Smoking, yes	5 (2.4)	5 (5.9)	–	0.049	3 (2.5)	3 (4.5)	4 (4.6)	0.711
Alcohol intake								
Never	57 (21.9)	20 (17.9)	26 (24.8)	0.458	44 (27.7)	22 (26.8)	31 (29.5)	0.912
Ever	203 (78.1)	92 (82.1)	79 (75.2)		115 (72.3)	60 (73.2)	74 (70.5)	
Physical activity level								
Low	148 (50.5)	71 (55.9)	60 (53.1)	0.788	88 (47.8)	47 (50.0)	69 (58.5)	0.036
Moderate	93 (31.7)	38 (29.9)	37 (32.7)		75 (40.8)	27 (28.7)	35 (29.7)	
High	52 (17.8)	18 (14.2)	16 (14.2)		21 (11.4)	20 (21.3)	14 (11.9)	
Body mass index, kg/m^2^	21.1 ± 1.2^a^	23.9 ± 0.6^b^	27.4 ± 2.6^c^	<0.001	21.2 ± 1.1^a^	24.0 ± 0.6^b^	27.2 ± 2.0^c^	<0.001
Waist circumference, cm	74.8 ± 4.4^a^	81.6 ± 4.1^b^	88.6 ± 7.1^c^	<0.001	75.5 ± 4.7^a^	81.6 ± 4.1^b^	88.5 ± 6.4^c^	<0.001
Total cholesterol, mg/dL	200.8 ± 33.7^a^	204.3 ± 33.8^ab^	214.8 ± 40.4^b^	0.002	216.3 ± 36.3	209.4 ± 35.8	209.3 ± 38.0	0.171
Triglycerides, mg/dL	85.7 ± 39.7^a^	99.5 ± 50.0^a^	124.8 ± 91.3^b^	<0.001	95.4 ± 44.2^a^	106.4 ± 60.2^a^	126.4 ± 66.2^b^	<0.001
HDL-cholesterol, mg/dL	68.1 ± 14.9^b^	59.8 ± 14.2^a^	57.4 ± 14.3^a^	<0.001	65.3 ± 16.2^b^	61.7 ± 14.5^ab^	57.2 ± 15.0^a^	<0.001
Fasting blood glucose, mg/dL	93.0 ± 9.3^a^	97.0 ± 22.4^ab^	100.3 ± 21.4^b^	<0.001	94.9 ± 13.7^a^	100.0 ± 21.2^b^	100.5 ± 14.0^b^	0.004

The data are presented as n(%) or mean ± standard deviation

Different letters represent statistical difference by Scheffe’s multiple comparison test

*P*-values were generated by analysis of variance followed by Scheffe’s multiple comparison test, or by Chi-squared test

Body mass index (*BMI*) was categorized as normal (18.5–22.9 kg/m^2^), overweight (23–24.9 kg/m^2^), or obese (≥25 kg/m^2^)

*HDL* high density lipoprotein, *KRW* Korean won

Table 2 shows the prevalence of 29 menopausal symptoms in three BMI subgroups in perimenopausal and postmenopausal groups. A woman was considered to have a particular symptom at the time of study if she gave a scores of 1 and 2–8 to the MENQOL symptoms (i.e., “1 for no symptom” and “2–8 for experienced symptom”). Both in perimenopause and postmenopause, obese women were significantly more likely than normal and overweight women to report having the vasomotor symptoms ‘hot flushes or flashes’ and ‘sweating’ and the physical symptoms ‘weight gain’ and ‘feeling bloated’.

**Table 2:** Prevalence of individual menopausal symptoms in normal, overweight, and obese perimenopausal and postmenopausal subjects

Components	Perimenopause				Postmenopause			
	Normal	Overweight	Obesity	*p*-value	Normal	Overweight	Obesity	*p*-value
	(*n* = 293)	(*n* = 127)	(*n* = 113)		(*n* = 184)	(*n* = 94)	(*n* = 118)	
Vasomotor symptoms, yes								
1. Hot flushes or flashes	137 (46.8)	51 (40.2)	66 (58.4)	0.017	113 (61.4)	56 (59.6)	87 (73.7)	0.046
2. Night sweats	96 (32.8)	35 (27.6)	36 (31.9)	0.567	84 (45.7)	41 (43.6)	63 (53.4)	0.292
3. Sweating	118 (40.3)	37 (29.1)	51 (45.1)	0.028	93 (50.5)	49 (52.1)	78 (66.1)	0.022
Psychosocial symptoms, yes								
4. Being dissatisfied with my personal life	145 (49.5)	64 (50.4)	53 (46.9)	0.852	108 (58.7)	56 (59.6)	79 (67.0)	0.328
5. Feeling anxious or nervous	181 (61.8)	74 (58.3)	63 (55.8)	0.506	133 (72.3)	63 (67.0)	82 (69.5)	0.649
6. Experiencing poor memory	244 (83.3)	103 (81.1)	92 (81.4)	0.828	161 (87.5)	83 (88.3)	111 (94.1)	0.167
7. Accomplishing less than I used to	227 (77.5)	98 (77.2)	88 (77.9)	0.991	148 (80.4)	77 (81.9)	98 (83.1)	0.845
8. Feeling depressed, down or blue	183 (62.5)	75 (59.1)	65 (57.5)	0.607	127 (69.0)	59 (62.8)	82 (69.5)	0.505
9. Being impatient with other people	179 (61.1)	73 (57.5)	71 (62.8)	0.676	128 (69.6)	58 (61.7)	81 (68.6)	0.394
10. Feeling of wanting to be alone	164 (56.0)	68 (53.5)	64 (56.6)	0.868	110 (59.8)	54 (57.5)	81 (68.6)	0.181
Physical symptoms, yes								
11. Flatulence (wind) or gas pains	168 (57.3)	77 (60.6)	67 (59.3)	0.807	111 (60.3)	56 (59.6)	71 (61.0)	0.978
12. Aching in muscles and joints	214 (73.0)	96 (75.6)	92 (81.4)	0.213	139 (75.5)	70 (74.5)	99 (83.9)	0.158
13. Feeling tired or worn out	255 (87.0)	103 (81.1)	95 (84.1)	0.281	157 (85.3)	78 (83.0)	98 (83.1)	0.822
14. Difficulty sleeping	165 (56.3)	62 (48.8)	67 (59.3)	0.223	124 (67.4)	57 (60.6)	79 (67.0)	0.501
15. Aches in back of neck or head	227 (77.5)	95 (74.8)	85 (75.2)	0.797	149 (81.0)	74 (78.7)	98 (83.1)	0.726
16. Decrease in physical strength	247 (84.3)	102 (80.3)	95 (84.1)	0.585	158 (85.9)	76 (80.9)	100 (84.8)	0.547
17. Decrease in stamina	283 (81.2)	105 (82.7)	91 (80.5)	0.905	158 (85.9)	74 (78.7)	94 (79.7)	0.223
18. Feeling a lack of energy	239 (81.6)	104 (81.9)	89 (78.8)	0.781	155 (84.2)	78 (83.0)	99 (83.9)	0.964
19. Dry skin	257 (87.7)	114 (89.8)	95 (84.1)	0.404	157 (85.3)	80 (85.1)	98 (83.1)	0.856
20. Weight gain	212 (72.4)	109 (85.8)	101 (89.4)	<0.001	129 (70.1)	82 (87.2)	106 (89.8)	<0.001
21. Increased facial hair	73 (24.9)	33 (26.0)	37 (33.6)	0.199	61 (33.2)	32 (34.0)	44 (37.3)	0.756
22. Changes in appearance, texture or tone of skin	240 (81.9)	103 (81.1)	92 (81.4)	0.979	165 (89.7)	79 (84.0)	98 (83.1)	0.198
23. Feeling bloated	180 (61.4)	91 (71.7)	88 (77.9)	0.003	121 (65.8)	65 (69.2)	94 (79.7)	0.033
24. Low backache	138 (47.1)	69 (54.3)	63 (55.8)	0.188	102 (55.4)	56 (59.6)	71 (60.2)	0.665
25. Frequent urination	201 (68.6)	80 (63.0)	80 (70.8)	0.388	137 (74.5)	72 (76.6)	85 (72.0)	0.749
26. Involuntary urination when laughing or coughing	198 (67.6)	90 (70.9)	84 (74.3)	0.395	124 (67.4)	63 (67.0)	85 (72.0)	0.644
Sexual symptoms, yes								
27. Change in sexual desire	188 (64.2)	86 (67.7)	83 (73.5)	0.200	148 (80.4)	71 (77.7)	86 (72.9)	0.308
28. Vaginal dryness during intercourse	183 (62.5)	78 (61.4)	73 (64.6)	0.873	155 (84.2)	77 (81.9)	89 (75.4)	0.157
29. Avoiding intimacy	191 (65.2)	83 (65.4)	78 (69.0)	0.752	147 (79.9)	74 (78.7)	89 (75.4)	0.651

The data are presented as n(%)

*P*-values were calculated using Chi-squared test

Body mass index (*BMI*) was categorized as normal (18.5–22.9 kg/m^2^), overweight (23–24.9 kg/m^2^), and obese (≥25 kg/m^2^)

The prevalence of each of the 29 individual component symptoms was determined by considering Menopause-Specific Quality of Life (MENQOL) scores of 1 and 2–8 to mean ‘no symptom’ and ‘experienced symptom’, respectively

Table 3 presents the prevalence of “moderate/severe” menopausal symptoms, as defined by a MENQOL domain score of ≥5. In perimenopause, obesity associated with a significantly higher prevalence of physical symptoms relative to normal weight or overweight. In postmenopause, obesity associated with a significantly higher prevalence of vasomotor symptoms.

**Table 3:** Prevalence of moderate/severe^a^ menopausal symptoms according to menopausal stage and body mass index

Domain	Perimenopause				Postmenopause			
	Normal	Overweight	Obesity	*p*-value	Normal	Overweight	Obesity	*p*-value
	(*n* = 293)	(*n* = 127)	(*n* = 113)		(*n* = 184)	(*n* = 94)	(*n* = 118)	
Total symptoms	24 (8.2)	8 (6.3)	16 (14.2)	0.081	35 (19.0)	18 (19.2)	26 (22.0)	0.795
Vasomotor	27 (9.2)	13 (10.2)	10 (8.9)	0.925	32 (17.4)	14 (14.9)	33 (28.0)	0.030
Psychosocial	40 (13.7)	15 (11.8)	19 (16.8)	0.527	39 (21.2)	23 (24.5)	26 (22.0)	0.823
Physical	47 (16.0)	19 (15.0)	29 (25.7)	0.048	28 (15.2)	21 (22.3)	29 (24.6)	0.104
Sexual	72 (24.6)	24 (18.9)	32 (28.3)	0.221	78 (42.4)	41 (43.6)	52 (44.1)	0.955

Body mass index (*BMI*) was categorized as normal (18.5–22.9 kg/m^2^), overweight (23–24.9 kg/m^2^), and obese (≥25 kg/m^2^)

*P*-values were calculated using Chi-squared test

^a^Moderate/severe menopausal symptom was defined as a Menopause-Specific Quality of Life (MENQOL) domain score of ≥5

Multiple linear regression analysis adjusted for age, education, parity, marital status, and physical activity showed that compared with normal, obesity associated with significantly more frequent physical symptoms in perimenopause and significantly more likely to have vasomotor symptoms in postmenopause. Similar associations were not detected for overweight. Moreover, BMI did not associate with psychosocial or experiencing of sexual symptoms (Table 4).

**Table 4:** Multiple linear regression analysis of the association between overweight and obesity and menopausal symptoms

Domain	Perimenopause					Postmenopause				
	Normal (*n* = 293)	Overweight (*n* = 127)		Obesity (*n* = 113)		Normal (*n* = 184)	Overweight (*n* = 94)		Obesity (*n* = 118)	
		β(S.E.)	*p*-value	β(S.E.)	*p*-value		β(S.E.)	*p*-value	β(S.E.)	*p*-value
Total symptoms										
Crude	ref.	−0.08 (0.14)	0.551	0.22 (0.15)	0.141	ref.	−0.02 (0.19)	0.934	0.22 (0.18)	0.216
Adjusted^a^		−0.09 (0.14)	0.543	0.21 (0.15)	0.152		0.06 (0.19)	0.751	0.26 (0.18)	0.148
Vasomotor										
Crude	ref.	−0.16 (0.17)	0.351	0.29 (0.18)	0.099	ref.	−0.05 (0.24)	0.824	0.63 (0.22)	0.005
Adjusted^a^		−0.19 (0.17)	0.253	0.23 (0.18)	0.196		0.01 (0.25)	0.958	0.68 (0.23)	0.003
Psychosocial										
Crude	ref.	−0.05 (0.16)	0.780	0.06 (0.17)	0.747	ref.	−0.01 (0.21)	0.964	0.18 (0.20)	0.369
Adjusted^a^		−0.06 (0.17)	0.731	0.05 (0.17)	0.780		0.05 (0.22)	0.824	0.18 (0.20)	0.368
Physical										
Crude	ref.	0.03 (0.15)	0.840	0.32 (0.15)	0.039	ref.	0.16 (0.18)	0.371	0.27 (0.17)	0.111
Adjusted^a^		0.04 (0.15)	0.787	0.35 (0.16)	0.023		0.21 (0.19)	0.270	0.29 (0.17)	0.090
Sexual										
Crude	ref.	−0.16 (0.20)	0.412	0.20 (0.21)	0.325	ref.	−0.16 (0.28)	0.552	−0.21 (0.26)	0.415
Adjusted^a^		−0.13 (0.20)	0.495	0.22 (0.21)	0.286		−0.02 (0.28)	0.936	−0.13 (0.25)	0.622

Body mass index (*BMI*) was categorized as normal (18.5–22.9 kg/m^2^), overweight (23–24.9 kg/m^2^), and obese (≥25 kg/m^2^)

^a^The regression analysis was adjusted for age (years), education (high school graduate or less, some college or more), parity (1–2, ≥3), marital status (yes or no answer to the question, “are you living with a spouse?”), and physical activity level (low, moderate, high)

## Discussion

This cross-sectional study of Korean middle-aged women showed that there were significant associations between obesity and menopausal symptoms. However, the associated symptom differed depending on menopausal stage: in perimenopause, obesity associated significantly with physical symptoms while, in postmenopause, obesity associated significantly with vasomotor symptoms. However, associations between overweight and menopausal symptoms were not detected.

Multiple observational studies have assessed the relationship between BMI or body fat and menopausal symptoms but the results are inconsistent. Two cross-sectional studies conducted in Australia and Turkey did not detect a relationship between BMI and menopausal symptoms among 45–60-year-old women [9, 10]. The Midlife Women’s Health Study also did not observe an association between BMI and hot flashes in pre- or perimenopausal women [19]. By contrast, a large observational study of 5968 postmenopausal Brazilian women suggested that obese women have more menopausal symptoms (as assessed using the Kupperman Menopausal Index) and that BMI may influence the intensity of vasomotor symptoms [27]. The Study of Women’s Health Across the Nation (SWAN) also showed significant associations between body fat and vasomotor symptoms, even after adjustment for confounding factors [12, 13]. Moreover, two recent interventional studies showed that weight loss can improve the hot flushes of obese or overweight midlife women; however, it should be noted that the studies had small sample sizes [14, 15]. The inconsistency of these study results may reflect various factors, including differences in study design, race/ethnicity, sample size, and environmental factors.

Many investigators have noted that vasomotor and physical symptoms are relatively common symptoms in menopausal period [28–30]. Indeed, in our study, the prevalence of hot flushes and night sweats were 54.9 and 38.2%, respectively. Moreover, many physical symptoms were very common; for example, 86.2% of the women reported having dry skin. These symptoms occur at different times of the menopausal period and can have a marked negative impact on the overall quality of women’s lives [3].

Despite the multiple studies that suggest obesity associates with menopausal symptoms (including ours), the pathophysiology behind this relationship is not yet clear [7, 11]. Two conflicting mechanisms have been proposed to explain the putative association between obesity and vasomotor symptoms. One proposes that obese women are simply more prone to vasomotor symptoms because adiposity or body fat acts as an insulator; obesity may inhibit dissipation of heat resulting from the increased internal body temperatures that arise from normal thermoregulatory mechanisms [7, 11]. The other mechanism proposes that obese women may have higher circulating estrogen levels because body fat is a source of estrogen and the peripheral conversion of androgens into estrogens takes place in adipose tissue; the high estrogen levels in obesity may decrease vasomotor symptoms. This mechanism has often been called the “thin hypothesis” [18, 31]. The present study is consistent with the first proposed mechanism: we showed that obesity associated significantly with more severe vasomotor symptoms. We also found that only obese, not overweight, women had significantly more menopausal symptoms than normal weight women. That obesity in menopause may be a risk factor for severe menopausal symptoms was also observed by other large-scale studies [12, 13, 27]. This observation is clinically significant because several studies recently reported that vasomotor symptoms during the menopause associate with a higher risk of adverse metabolic outcomes such as lower bone mineral density, insulin resistance, depression, and cardiovascular disease [32–35].

The multivariable analysis of our study also showed that during perimenopause, but not postmenopause, obese women were more likely than normal or overweight women to report moderate/severe physical symptoms, specifically weight gain and feeling bloated. Moreover, in postmenopause, but not perimenopause, obese women were more likely to report moderate/severe vasomotor symptoms, specifically hot flushes or flashes and sweating. The mechanisms underlying these relationships remain to be unclarified. Weight control may help improve these menopausal symptoms.

The present study has some limitations. Because this study had an observational cross-sectional design, the interpretation of direction of causality is debatable. Although our multivariable analysis showed that, in perimenopause and postmenopause, obesity associated with physical and vasomotor menopausal symptoms, respectively, our analysis of the prevalence of vasomotor and physical symptoms showed that obese women were more likely in general than normal women to report experiencing these symptoms regardless of menopausal stage. Thus, while weight gains during menopausal process may increase the menopausal symptoms, we cannot conclusively state that there is a direct causal relationship between weight gain and menopausal symptoms. Future prospective studies are needed to test a possible causal relationship. Second, although we adjusted our multivariable analysis for various confounders, it remains possible that there were other potential confounding factors that were not measured or are unknown. Finally, the generalizability of our study results to other women in Korea may be limited because only subjects who underwent a medical health check-up in a specific area of South Korea were recruited. This study feature may have resulted in inadvertent selection of a population that had a somewhat higher socio-economic status than general population.

Nevertheless, despite limitations discussed above, present study was the first to assess the association between BMI and menopausal symptoms at different stages of menopausal process in a Korea population. A major advantage of our study was that the menopausal symptom data were collected using self-administered MENQOL questionnaire, which is a validated instrument that has been used widely to examine the quality of life (including prevalence of menopausal symptoms) in middle-aged women.

## Conclusions

Our findings suggest that obesity in middle-aged Korean women associates positively with menopausal symptoms, although the associated symptoms differ depending on menopausal stage. This is clinically significant because several studies show that menopausal symptoms associate with adverse metabolic outcomes [31, 32, 35]. Since obesity is a well-known risk factor for chronic disease, these observations suggest that the health of middle-aged women may be improved by careful weight control: this may help to reduce both adverse metabolic outcomes and menopausal symptoms. Prospective studies that confirm our results and identify the underlying mechanisms are warranted.

## Additional files

Additional file 1: Flow chart of the subjects. (PPTX 36 kb)

Additional file 2: Questionnaire (the Korean translation version of MENQOL). (PDF 5456 kb)

## Abbreviations

BMI: Body mass index
HDL: High density lipoprotein
KRW: Korean won
MENQOL: Menopause-Specific Quality of Life
STRAW: Stages of Reproductive Aging Workshop
SWAN: Study of Women’s Health Across the Nation
WHO: World Health Organization
