# Supplementary material for: Obesity associates with vasomotor symptoms in postmenopause but with physical symptoms in perimenopause: a cross-sectional study
Source: BMC Womens Health. 2017 Dec 8;17:126. doi: 10.1186/s12905-017-0487-7 (PMC5721621; doi:10.1186/s12905-017-0487-7)
Supplement: Supplementary file 1 — Flow chart of the subjects. (PPTX 36 kb) [file 12905_2017_487_MOESM1_ESM.pptx]

## Slide 1
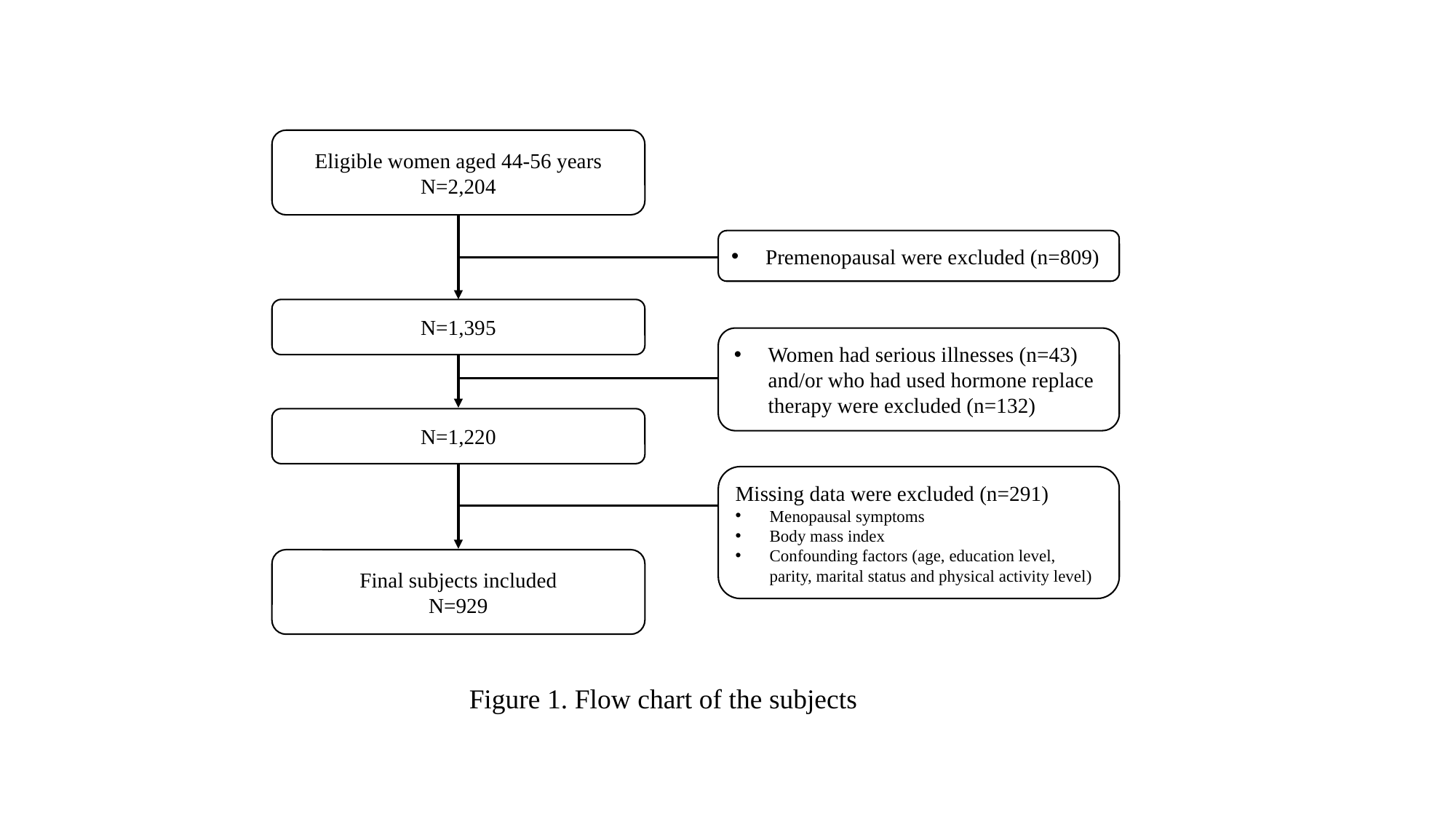

Eligible women aged 44-56 years N=2,204
Premenopausal were excluded (n=809)
N=1,395
Women had serious illnesses (n=43) and/or who had used hormone replace therapy were excluded (n=132)
N=1,220
Missing data were excluded (n=291)
Menopausal symptoms
Body mass index
Confounding factors (age, education level, parity, marital status and physical activity level)
Final subjects included
N=929
Figure 1. Flow chart of the subjects
