# Supplementary material for: Obesity associates with vasomotor symptoms in postmenopause but with physical symptoms in perimenopause: a cross-sectional study
Source: BMC Womens Health. 2017 Dec 8;17:126. doi: 10.1186/s12905-017-0487-7 (PMC5721621; doi:10.1186/s12905-017-0487-7)
Supplement: Supplementary file 2 — Questionnaire (the Korean translation version of MENQOL). (PDF 5456 kb) [file 12905_2017_487_MOESM2_ESM.pdf]

No. | | | |

# 갱년기(폐경기) 여성건강 실태조사

**다음 설문지는 만 45세-55세 사이의 중년 여성들에게 폐경기에 관한 인식 및 정보요구도, 증상, 지식, 태도, 관리에 대해 묻고 있습니다.**

**아래 용어 정의를 참조하시고, 각 질문에 대한 응답을 적절히 선택해 주시기 바랍니다.**

## 용어 정의

✓ **폐경:** 마지막 월경일로부터 1년이 지난 시점으로, 영구적으로 월경의 중단을 의미함

✓ **폐경기:** 폐경 주변기, 폐경 후를 통틀어서 말함

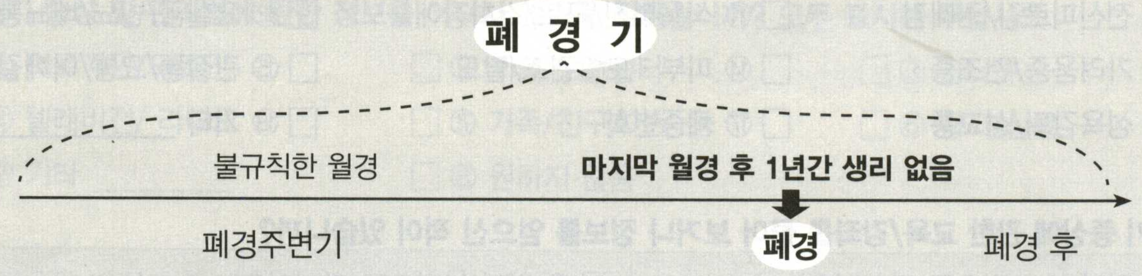


**1. 다음은 지난 1년간 월경(생리) 주기에 대한 질문입니다.**

지난 1년간 생리를 한적이 있습니까?

- ☐ 한번도 없었다 → 지난 1년간 임신, 출산이나 수유를 한적이 있습니까?
  - ☐ 1) 예 ☐ 2) 아니오
- ☐ 1번 이상 있었다
  - → 지난 1년간 7일 이상 차이가 나는 생리주기를 경험한 적이 있습니까?
    - ☐ 1) 예 _____번 ☐ 2) 아니오
  - → 지난 1년간 60일 이상 생리를 거른 적이 1번 이상 있습니까?
    - ☐ 1) 예 _____번 ☐ 2) 아니오

**2. 출산경험 (정상출산, 조산 등 모두 포함)이 있습니까?**

☐ 예 ☐ 아니오

**2-1. 첫 출산과 마지막 출산은 언제였습니까?**

첫 출산 연령 : 만 ☐☐ 세

마지막 출산 연령 만 ☐☐ 세

▣ 다음은 **폐경기 인식 및 정보 요구도**를 알아보고자 합니다. 각 문항을 잘 읽고 귀하가 알고 있는 대로 표시(✓)해 주십시오.

**1. 폐경에 대해 얼마나 걱정하십니까?**

□ ①전혀 걱정하지 않는다 □ ②걱정하지 않는다 □ ③걱정한다 □ ④ 매우 걱정한다

**2. 다음 중 폐경기 증상을 아는 대로 모두 표시해 주십시오**

□ ① 안면홍조 □ ② 손발에 열이 남 □ ③ 식은땀/발한
□ ④ 가슴답답함/두근거림 □ ⑤ 두통/어지러움 □ ⑥ 불안/초조/신경질
□ ⑦ 이명/난청 □ ⑧ 불면증/우울증 □ ⑨ 기억력 장애/건망증
□ ⑩ 전신피로감/권태감 □ ⑪ 식욕부진/구토/소화장애 □ ⑫ 요실금/빈뇨/배뇨통
□ ⑬ 가려움증/건조증 □ ⑭ 피부 • 모발건조/탈모 □ ⑮ 관절통/요통/어깨결림
□ ⑯ 성욕감퇴/성교통 □ ⑰ 체중변화 □ ⑱ 기타 ___________

**3. 폐경기 증상에 관한 교육/강좌를 들어 보거나 정보를 얻으신 적이 있습니까?**

□ ① 있다 (3-1번으로 가십시오) □ ② 없다 (4번으로 가십시오)

**3-1. 폐경기 증상에 관한 정보를 받으신 시기는 주로 언제입니까?**

□ ① 폐경 전 (현재 월경이 비교적 규칙적인 경우)
□ ② 폐경 주변기 (월경주기가 불규칙하면서 1년 이내 월경이 있는 경우)
□ ③ 폐경 후 (마지막 월경이 1년 이상 경과된 경우)

**3-2. 다음 매체 중 정보를 얻으신 경로를 모두 표시해 주십시오**

□ ① 의료기관/의료인 □ ② 지역 보건센터 □ ③ 인터넷
□ ④ 텔레비전/ 라디오 □ ⑤ 가족/친구/이웃 등
□ ⑥ 잡지/신문 □ ⑦ 기타___________

**3-3. 폐경기 증상에 관해 받으신 정보에 대해서 만족하셨습니까?**

□ ① 매우 불만족 □ ② 불만족 □ ③ 만족 □ ④ 매우 만족

**4. 폐경기 관련 정보 중 필요한 것을 모두 표시해 주십시오.**

☐ ① 폐경기 증상에 관한 정보

☐ ② 폐경과 관련된 주요질병에 관한 정보 (예. 골다공증, 심혈관 질환, 비만)

☐ ③ 폐경기관리에 도움이 되는 생활습관에 관한 정보 (예. 흡연, 음주, 체중조절, 수면 등)

☐ ④ 보완대체요법에 관한 정보(예. 단전호흡, 마사지, 한약, 영양제, 보조식품 등)

☐ ⑤ 호르몬 치료에 관한 정보

☐ ⑥ 폐경기 증후군 진단을 위한 검사에 관한 정보

☐ ⑦ 부부 성생활 관련 정보

☐ ⑧ 기타 ______________

☐ ⑨ 없음

**5. 폐경기 증상 및 관리법에 관한 정보를 어디서 얻기를 원하십니까? 모두 표시해 주십시오**

☐ ① 의료기관/의료인　☐ ② 지역 보건센터　☐ ③ 인터넷

☐ ④ 텔레비전/ 라디오　☐ ⑤ 가족/친구/이웃 등　☐ ⑥ 잡지/신문

☐ ⑦ 기타 __________　☐ ⑧ 원하지 않음

▣ 다음은 폐경기 증상의 치료법 중 하나인 **호르몬 치료**에 대한 질문입니다.
각 문항을 잘 읽고 귀하가 생각 하신 대로 표시( ✓ )해 주십시오.

**6. 폐경기 증상의 치료법 중 하나인 호르몬 치료에 대해 어느 정도 알고 있습니까?**

☐ ① 전혀 모른다　☐ ② 모른다　☐ ③ 알고 있다　☐ ④ 매우 잘 알고 있다

**7. 호르몬 치료에 대한 교육/강좌를 들어 보거나 정보를 얻은 적이 있습니까?**

☐ ① 있다 (7-1번으로 가십시오)　☐ ② 없다 (8번으로 가십시오)

**7-1. 호르몬 치료에 대한 정보를 얻으신 시기는 주로 언제입니까?**

☐ ① 폐경 전 (현재 월경이 비교적 규칙적인 경우)

☐ ② 폐경 주변기 (월경주기가 불규칙하면서 1년 이내 월경이 있는 경우)

☐ ③ 폐경 후 (마지막 월경이 1년 이상 경과된 경우)

**7-2. 다음 매체 중 호르몬 치료에 대한 정보를 얻으신 경로를 모두 표시해 주십시오**

☐ ① 의료기관/의료인　☐ ② 지역 보건센터　☐ ③ 인터넷

☐ ④ 텔레비전/ 라디오　☐ ⑤ 가족/친구/이웃 등　☐ ⑥ 잡지/신문

☐ ⑦ 기타__________

**7-3. 받으신 정보에 대해서 만족하셨습니까?**

☐ ① 매우 불만족　☐ ② 불만족　☐ ③ 만족　☐ ④ 매우 만족

▣ 다음은 폐경기 증상의 치료법 중 하나인 **호르몬 치료**에 대한 질문입니다.
각 문항을 잘 읽고 귀하가 생각 하신 대로 표시( ✓ )해 주십시오.

| | 문 항 | 전혀 그렇지 않다 | 그렇지 않다 | 그렇다 | 매우 그렇다 |
|---|---|---|---|---|---|
| 8 | 폐경기 증상완화를 위한 호르몬 치료가 필요하다고 생각하십니까? | 1 | 2 | 3 | 4 |
| 9 | 호르몬 치료가 폐경 관련 **열성, 홍조, 발한, 불면증**을 관리하는데 도움이 될 것이라고 생각하십니까? | 1 | 2 | 3 | 4 |
| 10 | 호르몬 치료가 폐경 관련 **질건조, 피부건조, 빈뇨증상**을 관리하는데 도움이 될 것이라고 생각하십니까? | 1 | 2 | 3 | 4 |
| 11 | 호르몬 치료가 **골다공증 예방**에 도움이 될 것이라고 생각하십니까? | 1 | 2 | 3 | 4 |
| 12 | 호르몬 치료가 **심혈관 질환 및 예방**에 도움이 될 것이라고 생각하십니까? | 1 | 2 | 3 | 4 |
| 13 | 호르몬 치료를 받으면 **유방암에 걸릴 위험**이 커질 것이라고 생각하십니까? | 1 | 2 | 3 | 4 |

▣ **다음은 중년여성들에게 나타날 수 있는 증상입니다.**
각 문항마다 귀하와 가장 가깝다고 생각하는 정도에 표시( ✓ )해 주십시오.

| | 문 항 | 증상 없음 | 증상이 있는 경우 | | | | | | |
|---|---|---|---|---|---|---|---|---|---|
| | | | 전혀 괴롭지 않다 ←→ 너무 괴롭다 | | | | | | |
| 1 | 얼굴이 화끈거린다 | ☐ | 0 | 1 | 2 | 3 | 4 | 5 | 6 |
| 2 | 밤에 땀이 난다 | ☐ | 0 | 1 | 2 | 3 | 4 | 5 | 6 |
| 3 | 땀이 많이 난다 | ☐ | 0 | 1 | 2 | 3 | 4 | 5 | 6 |
| 4 | 생활이 만족스럽지 못하다 | ☐ | 0 | 1 | 2 | 3 | 4 | 5 | 6 |
| 5 | 불안하거나 짜증이 난다 | ☐ | 0 | 1 | 2 | 3 | 4 | 5 | 6 |
| 6 | 기억력이 떨어진다 | ☐ | 0 | 1 | 2 | 3 | 4 | 5 | 6 |
| 7 | 일하는 것이 예전 같지 않다 | ☐ | 0 | 1 | 2 | 3 | 4 | 5 | 6 |

| 문 항 | | 증상 없음 | 증상이 있는 경우<br>전혀 괴롭지 않다 ←→ 너무 괴롭다 | | | | | | |
|---|---|---|---|---|---|---|---|---|---|
| 8 | 침울하고 우울하다 | □ | 0 | 1 | 2 | 3 | 4 | 5 | 6 |
| 9 | 인간관계에서 참을성이 부족해진다 | □ | 0 | 1 | 2 | 3 | 4 | 5 | 6 |
| 10 | 혼자 있고 싶다 | □ | 0 | 1 | 2 | 3 | 4 | 5 | 6 |
| 11 | 배에 가스가 많이 차거나 아프다 | □ | 0 | 1 | 2 | 3 | 4 | 5 | 6 |
| 12 | 근육이나 관절이 아프다 | □ | 0 | 1 | 2 | 3 | 4 | 5 | 6 |
| 13 | 자주 피곤하고 쉽게 지친다 | □ | 0 | 1 | 2 | 3 | 4 | 5 | 6 |
| 14 | 잠을 자기가 어렵다 | □ | 0 | 1 | 2 | 3 | 4 | 5 | 6 |
| 15 | 등, 뒷목 또는 머리가 아프다 | □ | 0 | 1 | 2 | 3 | 4 | 5 | 6 |
| 16 | 근력이 떨어진다 | □ | 0 | 1 | 2 | 3 | 4 | 5 | 6 |
| 17 | 지구력이 약해진다 | □ | 0 | 1 | 2 | 3 | 4 | 5 | 6 |
| 18 | 활력이 떨어짐을 느낀다 | □ | 0 | 1 | 2 | 3 | 4 | 5 | 6 |
| 19 | 피부가 건조하다 | □ | 0 | 1 | 2 | 3 | 4 | 5 | 6 |
| 20 | 살이 찐다 | □ | 0 | 1 | 2 | 3 | 4 | 5 | 6 |
| 21 | 얼굴에 털이 난다 | □ | 0 | 1 | 2 | 3 | 4 | 5 | 6 |
| 22 | 외모, 피부결 또는 피부의 탄력이 변한다 | □ | 0 | 1 | 2 | 3 | 4 | 5 | 6 |
| 23 | 붓는 느낌이 든다 | □ | 0 | 1 | 2 | 3 | 4 | 5 | 6 |
| 24 | 요통이 있다 | □ | 0 | 1 | 2 | 3 | 4 | 5 | 6 |
| 25 | 소변을 자주 본다 | □ | 0 | 1 | 2 | 3 | 4 | 5 | 6 |
| 26 | 웃거나 기침을 할 때 나도 모르게 소변이 새어 나온다 | □ | 0 | 1 | 2 | 3 | 4 | 5 | 6 |
| 27 | 성욕에 변화가 있다 | □ | 0 | 1 | 2 | 3 | 4 | 5 | 6 |
| 28 | 부부생활 시 질이 건조하다 | □ | 0 | 1 | 2 | 3 | 4 | 5 | 6 |
| 29 | 부부생활을 피하게 된다 | □ | 0 | 1 | 2 | 3 | 4 | 5 | 6 |

▣ 다음은 **<u>폐경기에 관한 설명</u>**입니다. 각 문항을 잘 읽고 귀하가 알고 있는 대로 표시( ✓ )해 주십시오.

| | 문항 | 예 | 아니오 | 모르겠다 |
|---|---|---|---|---|
| 1 | 폐경기 동안 난소에서는 여성호르몬 분비가 증가한다. | ☐ | ☐ | ☐ |
| 2 | 대부분의 여성은 폐경기 동안 불편함을 경험하지 않는다. | ☐ | ☐ | ☐ |
| 3 | 폐경기는 평균연령 45세에 온다. | ☐ | ☐ | ☐ |
| 4 | 에스트로겐 호르몬의 사용은 일반적으로 폐경의 증상을 없앤다. | ☐ | ☐ | ☐ |
| 5 | 55세가 되면 모든 여성은 폐경이 된다. | ☐ | ☐ | ☐ |
| 6 | 폐경기 동안 외부 생식기는 조여들고 질 분비물이 마른다. | ☐ | ☐ | ☐ |
| 7 | 폐경기 동안 월경량은 줄어들고 월경일수가 점점 짧아진다. | ☐ | ☐ | ☐ |
| 8 | 폐경기 동안 월경이 불규칙해진다. | ☐ | ☐ | ☐ |
| 9 | 폐경기 동안 어떤 여성들은 불안, 흥분, 우울, 신경과민증을 경험한다. | ☐ | ☐ | ☐ |
| 10 | 폐경 때문에 정신이상에 걸릴 위험성이 훨씬 높다. | ☐ | ☐ | ☐ |
| 11 | 폐경 이후에는 골다공증이 올 가능성이 증가한다. | ☐ | ☐ | ☐ |
| 12 | 폐경 이후에는 심혈관 질환에 걸릴 가능성이 증가한다. | ☐ | ☐ | ☐ |
| 13 | 폐경 이후에는 더 이상 임신할 수 없다. | ☐ | ☐ | ☐ |
| 14 | 화끈거림, 얼굴, 목, 가슴에서 흐르는 식은땀은 폐경기 중에 일어나는 가장 흔한 증상이다. | ☐ | ☐ | ☐ |
| 15 | 폐경 이후에는 배뇨장애, 요실금 등이 일어날 수 있다. | ☐ | ☐ | ☐ |
| 16 | 폐경 이후에는 관절통, 허리통증이 일어날 수 있다. | ☐ | ☐ | ☐ |

▣ 다음은 귀하의 **폐경에 대한 태도**를 알아보고자 하는 문항입니다.
아래를 읽고 귀하의 생각과 가장 가깝다고 생각하는 정도에 표시( ✓ )해 주십시오.

| 문항 | | 전혀 그렇지 않다 | 그렇지 않다 | 조금 그렇다 | 매우 그렇다 |
|---|---|---|---|---|---|
| 1 | 여성들에게 폐경은 유쾌하지 않은 경험이다 | □ | □ | □ | □ |
| 2 | 여성들이 폐경을 싫어하는 것은 당연하다 | □ | □ | □ | □ |
| 3 | 폐경이 되면 폐경에 따른 문제가 있을 것이라고 생각한다 | □ | □ | □ | □ |
| 4 | 폐경경험은 여성들을 혼란스럽게 만든다고 생각한다 | □ | □ | □ | □ |
| 5 | 폐경이 되면 여성들은 의기소침해질 것이라고 생각한다 | □ | □ | □ | □ |
| 6 | 폐경이 되면 여성들은 우울해질 것이라고 생각한다 | □ | □ | □ | □ |
| 7 | 폐경이 되면 여성들은 폐경전보다 기분이 좋아질 것이라 생각한다 | □ | □ | □ | □ |
| 8 | 폐경을 경험하면서 여성들은 인생에 대해 더 넓은 견해를 갖게 될 것이라 생각한다 | □ | □ | □ | □ |
| 9 | 폐경이 되면 여성들은 자신감이 커질 것이라 생각한다 | □ | □ | □ | □ |
| 10 | 폐경이 되면 여성들은 폐경전보다 평온하고 행복해질 것이라 생각한다 | □ | □ | □ | □ |
| 11 | 폐경이 되면 여성들은 인생이 더 살맛나게 느껴질 것이라 생각한다 | □ | □ | □ | □ |
| 12 | 폐경이 되면 여성들은 자기 자신을 위해 더 자유롭게 일을 할 것이라 생각한다 | □ | □ | □ | □ |
| 13 | 폐경후 여성들은 남편과의 관계가 더 좋아질 것이라 생각한다 | □ | □ | □ | □ |
| 14 | 여성들은 폐경을 지금까지 자신에게 일어난 일 중 가장 좋은 일이라고 생각할 것이라 생각한다 | □ | □ | □ | □ |

| | 문항 | 전혀 그렇지 않다 | 그렇지 않다 | 조금 그렇다 | 매우 그렇다 |
|---|---|---|---|---|---|
| 15 | 폐경이 되면 여성들은 전보다 주변 일에 더 관심을 갖게 될 것이라 생각한다 | ☐ | ☐ | ☐ | ☐ |
| 16 | 폐경으로 몸의 변화가 있기는 하지만 그렇게 많은 변화가 있는 것은 아니라 생각한다 | ☐ | ☐ | ☐ | ☐ |
| 17 | 폐경전과 폐경후의 차이는 단지 월경을 하느냐 안 하느냐의 차이일 뿐 다른 의미는 없다고 생각한다. | ☐ | ☐ | ☐ | ☐ |
| 18 | 폐경이 되는 것은 여성에게 그렇게 중요한 측면이 변하는 것은 아니라 생각한다 | ☐ | ☐ | ☐ | ☐ |
| 19 | 폐경으로 고통을 겪는 여성들은 보통 시간이 많은 한가한 사람들이라고 생각한다 | ☐ | ☐ | ☐ | ☐ |
| 20 | 폐경으로 고통을 겪는 여성들은 평소에 폐경이 되면 어려움이 있을 것이라고 생각해 온 사람들이라 생각한다 | ☐ | ☐ | ☐ | ☐ |
| 21 | 폐경으로 인한 고통은 여성 스스로가 신체 변화에 대한 통제력이 없다는 것을 의미한다고 생각한다 | ☐ | ☐ | ☐ | ☐ |
| 22 | 폐경이 되면 여성들은 자기중심적이 된다고 생각한다 | ☐ | ☐ | ☐ | ☐ |
| 23 | 폐경이 되면 대부분의 여성들은 이제 나는 진짜 여자가 아니라는 생각을 한다 | ☐ | ☐ | ☐ | ☐ |
| 24 | 여성들은 폐경이 된 것을 사람들의 관심을 끌기 위한 하나의 구실로 사용한다고 생각한다 | ☐ | ☐ | ☐ | ☐ |
| 25 | 폐경이 되면 여성들은 허전함을 느낄까봐 걱정할 것이라고 생각한다 | ☐ | ☐ | ☐ | ☐ |
| 26 | 폐경은 여성으로 하여금 자신도 이해할 수 없는 이상한 행동을 하게 하는 경향이 있다고 생각한다 | ☐ | ☐ | ☐ | ☐ |
| 27 | 폐경경험은 여성들이 이해하기 어려운 신비로운 일이라고 생각한다 | ☐ | ☐ | ☐ | ☐ |

▣ 다음은 **폐경기관리와 관련된 호르몬치료 및 보완대체요법**에 대해 알아보고자 하는 문항입니다.

**1. 폐경기에 접어들면서 불편을 느끼는 점을 모두 표시해 주십시오.**

☐ ① 안면홍조 등 다양한 신체적 증상
☐ ② 신경질, 불면증, 우울증 등 정신적 증상
☐ ③ 원활하지 못한 부부 성생활
☐ ④ 다양한 만성질환 발생
☐ ⑤ 향후 치매 발병에 대한 우려
☐ ⑥ 기억력과 집중력 저하
☐ ⑦ 가정/사회에서 역할변화 적응의 어려움
☐ ⑧ 기타 ____________
☐ ⑨ 해당 없음

**2. 폐경기 증상으로 인해 병원에 방문한 적이 있습니까?**

☐ ① 있다 (☞ 2-1번으로 가십시오)
☐ ② 없다 (☞ 3번으로 가십시오)

**2-1. 만일 있다면 어느 과로 내원하셨습니까? (모두 표시해 주십시오)**

☐ ① 산부인과
☐ ② 가정의학과
☐ ③ 내분비 내과
☐ ④ 피부과
☐ ⑤ 비뇨기과
☐ ⑥ 정신건강 의학과
☐ ⑦ 한방병원
☐ ⑧ 기타 ____________

**2-2. 병원 방문 당시 있었던 주요 증상을 모두 표시해 주십시오**

☐ ① 안면홍조
☐ ② 손발에 열이 남
☐ ③ 식은땀/발한
☐ ④ 가슴답답함/두근거림
☐ ⑤ 두통/어지러움
☐ ⑥ 불안/초조/신경질
☐ ⑦ 이명/난청
☐ ⑧ 불면증/우울증
☐ ⑨ 기억력 장애/건망증
☐ ⑩ 전신피로감/권태감
☐ ⑪ 식욕부진/구토/소화장애
☐ ⑫ 요실금/빈뇨/배뇨통
☐ ⑬ 가려움증/건조증
☐ ⑭ 피부 · 모발건조/탈모
☐ ⑮ 관절통/요통/어깨결림
☐ ⑯ 성욕감퇴/성교통
☐ ⑰ 체중변화
☐ ⑱ 기타 ____________

**3. 폐경기 증상완화를 목적으로 호르몬 치료를 의료진으로부터 권유 받은 적이 있습니까?**

☐ ① 예
☐ ② 아니오

**4. 폐경기 증상완화를 목적으로 호르몬 치료를 받으신 적이 있습니까?**

☐ ① 예 (☞ 4-1번으로 가십시오)
☐ ② 아니오 (☞ 5번으로 가십시오)

**4-1. 어느 진료과로부터 처방을 받았습니까? (모두 표시해 주십시오)**

- ☐ ① 산부인과
- ☐ ② 가정의학과
- ☐ ③ 내분비 내과
- ☐ ④ 피부과
- ☐ ⑤ 비뇨기과
- ☐ ⑥ 정신건강 의학과
- ☐ ⑦ 한방병원
- ☐ ⑧ 기타 ____________

**4-2. 의사는 호르몬치료를 어떻게 설명하셨습니까?**

- ☐ ① 부작용보다 이점에 대한 설명을 주로 하였다.
- ☐ ② 이점보다는 부작용에 대한 설명을 주로 하였다.
- ☐ ③ 부작용과 이점을 동일하게 설명하였다.
- ☐ ④ 구체적으로 설명 하지 않았다.
- ☐ ⑤ 기타 ____________

**4-3. 현재도 호르몬 치료를 받고 계십니까?** ☐ ① 예 ☐ ② 아니오

**4-4. 처음으로 호르몬 치료를 시작한 시기는 언제입니까?** 만 ____________세

**4-5. 호르몬 치료는 얼마 동안 받으셨습니까?** __________년 __________개월

**4-6. 다음 중 사용하셨던 호르몬 형태를 모두 표시해 주십시오**

- ☐ ① 경구용
- ☐ ② 주사제
- ☐ ③ 크림 또는 겔
- ☐ ④ 패치

**4-7. 호르몬 치료가 폐경증상 완화 및 전반적인 건강 증진에 도움이 되었다고 생각하십니까?**

- ☐ ① 전혀 도움이 되지 않았다
- ☐ ② 도움이 되지 않았다
- ☐ ③ 도움이 되었다
- ☐ ④ 매우 도움이 되었다

**4-8. 호르몬 치료 후 완화된 증상을 모두 표시해 주십시오**

- ☐ ① 안면홍조
- ☐ ② 손발에 열이 남
- ☐ ③ 식은땀/발한
- ☐ ④ 가슴답답함/두근거림
- ☐ ⑤ 두통/어지러움
- ☐ ⑥ 불안/초조/신경질
- ☐ ⑦ 이명/난청
- ☐ ⑧ 불면증/우울증
- ☐ ⑨ 기억력 장애/건망증
- ☐ ⑩ 전신피로감/권태감
- ☐ ⑪ 식욕부진/구토/소화장애
- ☐ ⑫ 요실금/빈뇨/배뇨통
- ☐ ⑬ 가려움증/건조증
- ☐ ⑭ 피부 · 모발건조/탈모
- ☐ ⑮ 관절통/요통/어깨결림
- ☐ ⑯ 성욕감퇴/성교통
- ☐ ⑰ 체중변화
- ☐ ⑱기타 ____________

**4-9. 호르몬 치료를 중단 하셨다면, 중단 이유는 무엇입니까? 모두 표시해 주십시오**

- ☐ ① 부작용 때문에
- ☐ ② 증상이 호전되어서
- ☐ ③ 효과가 없는 것 같아서
- ☐ ④ 비용 때문에
- ☐ ⑤ 기타 ____________

**5. (☞ 4 번 문항에서 '아니오'를 표시하신 분만) 폐경기에 호르몬 치료를 받지 않은 이유는 무엇입니까?**
**모두 표시해 주십시오**

☐ ① 부작용이 걱정되어서　☐ ② 효과가 없을 것 같아서　☐ ③ 정보가 부족해서
☐ ④ 건강관리에 관심이 없어서　☐ ⑤ 증상이 거의 없어서　☐ ⑥ 비용 때문에
☐ ⑦ 기타 ____________

**6. 최소한 한달 이상 폐경증상 예방 및 완화를 위해 보완대체요법을 사용해 보신적이 있습니까?**

☐ 예 (☞ 6-1번으로 가십시오)　☐ 아니오 (☞ 7번으로 가십시오)

**6-1. 사용해 본 적 있는 보완대체요법을 모두 표시(v)해 주십시오**

☐ 운동요법　☐ 마사지　☐ 단전호흡 (예: 명상, 요가)
☐ 향기요법 (아로마테라피)　☐ 한방요법 (예:침술, 한약)　☐ 심리치료
☐ 천연 식물성 호르몬제　☐ 태반주사 요법　☐ 기타 ____________
☐ 비타민, 영양제 →

**6-2. 복용해 본 적 있는 비타민, 영양제를 모두 표시(v)해 주십시오**

☐ 종합 비타민 및 무기질 제품　☐ 오메가-3 지방산　☐ 칼슘
☐ 글루코사민　☐ 감마리놀렌산　☐ 엽산
☐ 철분제　☐ 비타민 B　☐ 비타민 C
☐ 비타민 D　☐ 비타민 E　☐ 코엔자임 큐
☐ 기타 ____________

☐ 건강기능 식품 →

**6-3. 복용해 본 적 있는 건강기능 식품을 모두 표시(v)해 주십시오**

☐ 이소플라본 (콩 식품 포함)　☐ 달맞이꽃 종자유　☐ 로얄제리
☐ 홍삼　☐ 인삼　☐ 석류 및 석류음료
☐ 아마씨 오일　☐ 칡즙　☐ 알로에 제품
☐ 클로렐라　☐ 각종 차 (허브차, 녹차, 홍차)
☐ 백수오　☐ 기타 ____________

**6-4. 보완대체요법을 사용하게 된 계기를 모두 표시해 주십시오**

☐ ① 가족/친구/이웃의 추천 ☐ ② 인터넷 검색을 통해 ☐ ③ 텔레비전을 통해
☐ ④ 라디오를 통해 ☐ ⑤ 잡지/신문을 통해 ☐ ⑥ 의사 추천
☐ ⑦ 한의사 추천 ☐ ⑧ 약사 추천 ☐ ⑨ 영양사 추천
☐ ⑩ 자신이 원해서 ☐ ⑪ 기타 ____________

**6-5. 보완대체요법이 폐경 증상 완화 및 전반적인 건강 증진에 도움이 되었다고 생각 하십니까?**

☐ ① 전혀 그렇지 않다 ☐ ② 그렇지 않다 ☐ ③ 그렇다 ☐ ④ 매우 그렇다

**6-6. 사용 전 보완대체요법에 대한 효용과 안전성에 대한 정보를 충분히 얻었습니까?**

☐ ① 전혀 그렇지 않다 ☐ ② 그렇지 않다 ☐ ③ 그렇다 ☐ ④ 매우 그렇다

**6-7. 만약 사용을 중단 하셨다면, 중단 이유는 무엇입니까? 모두 표시해 주십시오**

☐ ① 부작용 때문에 ☐ ② 증상이 호전되어서 ☐ ③ 효과가 없는 것 같아서
☐ ④ 비용 때문에 ☐ ⑤ 기타 ____________

**7. (☞ 6번 문항에서 '아니오' 를 표시 하신 분만) 폐경기 관리를 위해 보완대체요법을 사용하지 않았다면, 그 이유는 무엇입니까? 모두 표시해 주십시오**

☐ ① 부작용이 걱정되어서 ☐ ② 효과가 없을 것 같아서 ☐ ③ 정보가 부족해서
☐ ④ 건강관리에 관심이 없어서 ☐ ⑤ 증상이 거의 없어서 ☐ ⑥ 비용 때문에
☐ ⑦ 기타 ____________

## ♣ 설문에 참여해주셔서 대단히 감사합니다 ♣
